# Is CT-Based Perfusion and Collateral Imaging Sensitive to Time Since Stroke Onset?

**DOI:** 10.3389/fneur.2015.00070

**Published:** 2015-04-09

**Authors:** Smriti Agarwal, Tomasz Matys, S. Tulasi Marrapu, Daniel J. Scoffings, Jennifer Mitchell, P. Simon Jones, Jean-Claude Baron, Elizabeth A. Warburton

**Affiliations:** ^1^Clinical Neurosciences, University of Cambridge, Cambridge, UK; ^2^Department of Radiology, Addenbrooke’s Hospital, Cambridge, UK; ^3^Stroke Unit, Addenbrooke’s Hospital, Cambridge, UK; ^4^University of Cambridge, Cambridge, UK; ^5^Centre de Psychiatrie et Neurosciences, INSERM U894, Hôpital Sainte-Anne, Université Paris 5, Paris, France

**Keywords:** CT perfusion, collaterals, stroke, time, onset

## Abstract

**Purpose:**

CT-based perfusion and collateral imaging is increasingly used in the assessment of patients with acute stroke. Time of stroke onset is a critical factor in determining eligibility for and benefit from thrombolysis. Animal studies predict that the volume of ischemic penumbra decreases with time. Here, we evaluate if CT is able to detect a relationship between perfusion or collateral status, as assessed by CT, and time since stroke onset.

**Materials and methods:**

We studied 53 consecutive patients with proximal vessel occlusions, mean (SD) age of 71.3 (14.9) years, at a mean (SD) of 125.2 (55.3) minutes from onset, using whole-brain CT perfusion (CTp) imaging. Penumbra was defined using voxel-based thresholds for cerebral blood flow (CBF) and mean transit time (MTT); core was defined by cerebral blood volume (CBV). Normalized penumbra fraction was calculated as Penumbra volume/(Penumbra volume + Core volume) for both CBF and MTT (Pen_CBF_ and Pen_MTT_, respectively). Collaterals were assessed on CT angiography (CTA). CTp ASPECTS score was applied visually, lower scores indicating larger lesions. ASPECTS ratios were calculated corresponding to penumbra fractions.

**Results:**

Both Pen_CBF_ and Pen_MTT_ showed decremental trends with increasing time since onset (Kendall’s tau-*b* = −0.196, *p* = 0.055, and −0.187, *p* = 0.068, respectively). The CBF/CBV ASPECTS ratio, which showed a relationship to Pen_CBF_ (Kendall’s tau-*b* = 0.190, *p* = 0.070), decreased with increasing time since onset (Kendall’s tau-*b* = −0.265, *p* = 0.006). Collateral response did not relate to time (Kendall’s tau-*b* = −0.039, *p* = 0.724).

**Conclusion:**

Even within 4.5 h since stroke onset, a decremental relationship between penumbra and time, but not between collateral status and time, may be detected using perfusion CT imaging. The trends that we demonstrate merit evaluation in larger datasets to confirm our results, which may have potential wider applications, e.g., in the setting of strokes of unknown onset time.

## Introduction

CT-based perfusion and collateral imaging is increasingly used in the assessment of patients with acute stroke (1–6). Thus, CT perfusion (CTp) is used to identify core and penumbra by mapping cerebral blood flow (CBF), cerebral blood volume (CBV), and mean transit time (MTT) (1, 7). CBF (2) and MTT (2, 8, 9) thresholds have been used to identify penumbral tissue, while CBV (9) has been used for mapping the infarct core. While there have been a number of studies demonstrating that CTp may be used to identify these tissue compartments, there is some disagreement in the literature about its utility in routine clinical practice to guide early treatment decisions (10). However, its wider availability, shorter scanning times, and lower costs (11) make it potentially more attractive as a clinical tool than MRI which is an expensive and time intensive resource, and may not be available 24 hours a day at many institutions for the assessment of acute stroke.

Time of stroke onset is a critical factor in determining eligibility for and benefit from thrombolysis (12). From a pathophysiological perspective, ischemic penumbra is the therapeutic target for acute stroke therapies (1, 13, 14). An important concept relating to the penumbra remains that, unless salvaged, it gets recruited to the ischemic core with time (15). While PET remains the gold standard for penumbral imaging (14, 15), MRI, and CT-based methods have been applied in clinical cohorts to study penumbral tissue and relationship with clinical outcomes (16, 17). Pre-clinical studies and few human imaging based studies report a falling frequency of target mismatch as a surrogate for penumbral tissue with increasing time from ictus (18). We hypothesized that within the therapeutic window for thrombolysis, a decremental relationship of penumbra with time since stroke onset could be applied using perfusion CT imaging in a clinical population. The further aim of establishing such a relationship would be in the setting where time of stroke onset is unknown, and physiological imaging may have a role (1, 19). Physiological imaging also has potential for extending the time window for early treatments beyond 4.5 h, and some successful studies including patients up to 6 h have already emerged (20, 21).

We studied a cohort of patients with whole head CTp imaging within the currently licensed window for thrombolysis, namely 4.5 h. We examined voxel-based quantitative tissue fractions against time since onset. CTp thresholds have been shown to be robust in that, they are not time dependent within this window and potentially up to 15 h from ictus (22); and can thus be reliably applied in this early time window. Given the hemodynamic changes after an acute vascular occlusion, i.e., falling CBF and subsequently CBV (15), these two parameters alongside MTT were applied to quantitatively describe the penumbra. To further explore a clinically translatable index, visually assessed ASPECTS score for CTp maps, as previously described in the literature (23, 24), was examined against quantitative fractions and further, against time since onset.

We aimed to show that CTp is a reasonable imaging modality to capture these expected tissue changes, given the current uncertainties regarding its utility, as outlined above. Relationship of penumbra and time has been studied previously in physiological imaging studies (16, 18) and we aimed to demonstrate that CTp imaging may be applied in a clinical population to confirm these expected relationships. In addition, given the potential role of collateral status in maintaining the penumbra (25–27), collateral circulation was also examined against time. This is an important area of current research and such a relationship has not, to our knowledge, been previously assessed.

## Materials and Methods

### Approvals

We recruited patients from the Cambridge Acute Stroke Database. Ethical approval was granted by the South East Research Ethics Committee and the National Information Governance Board (NIGB, UK). Patients or next of kin provided written informed consent. When consent was unavailable, approval was in place for retrieval of clinical and imaging data.

### Patient recruitment

A cohort of consecutive anterior circulation stroke patients (*n* = 53) with proximal arterial occlusions, i.e., the intracranial internal carotid artery (ICA) or proximal middle cerebral artery (MCA) (M1), confirmed on CTA, was recruited between December 2009 and February 2013. We selected proximal occlusions to avoid including patients where recanalization had already occurred; thus, penumbral tissue could be reliably studied. All patients had presented within 4.5 h of clearly known onset of symptoms, were being assessed for thrombolysis and underwent CTp as part of the acute stroke imaging protocol at our institution. All patients had presented with their first clinical stroke and had a clear defect on CTp. Lacunar strokes were excluded because penumbral tissue characterization is unclear in these cases (28). Baseline clinical data were recorded prospectively.

### Whole-brain CT perfusion imaging acquisition and analysis

Plain CT and CTp were acquired in succession using Siemens^®^ Somatom Definition Flash Scanner. Perfusion images were acquired after a 4 s delay following an injection of 50 ml of Niopam-300 with a PSI injector at a rate of 5 ml/s and a saline chaser bolus, via a 16–18 gage intravenous cannula. *Z*-axis coverage was 70–100 mm, with acquisition parameters of 80 kV and 240–250 mA, rotation time of 0.28–1 s, and reconstructed slice thickness of 10 mm with 4–6 mm overlap.

Raw perfusion data were analyzed on a Siemens^®^ workstation using Syngo^®^ VPCT *Neuro* software. Brain parenchyma was isolated by skull bone contour findings; CSF and calcifications were removed by automatic thresholding. The arterial input function (AIF) and venous outflow function were semi-automatically selected from the anterior cerebral artery (ACA) and superior sagittal sinus respectively. In two cases, the MCA was used to derive AIF. Major vessels were removed by applying relative thresholding to the maximal voxel enhancement. Adaptive spatial filtering was performed that did not smooth over edges and vessel borders. Subsequently, quantitative maps of relative CBF, CBV, and MTT were obtained using a deconvolution algorithm.

Quantitative maps were transferred to a Windows^®^ PC and segmented using voxel-based thresholds to define at-risk tissue or “penumbra” and irreversibly damaged tissue or “core” (3). This was performed using in-house software (3, 17) in Matlab^®^ (R2007b, The MathWorks Inc.) run using SPM8 (Wellcome Trust Centre for Imaging Neuroscience, London, UK).

### Penumbra fraction definitions

Penumbra was defined using a previously validated, voxel-based quantitative threshold based for CBF [volume of tissue where each voxel had a CBF ratio of affected to unaffected hemisphere (A/U) ≤0.50 outside the core] based on large clinical series (2). For further substantiation, we also applied a threshold based on MTT (9, 29, 30) (volume of tissue where each voxel had an MTT ratio of A/U ≥1.45 outside the core). Ischemic core was defined using a CBV threshold [volume of tissue where each voxel had a CBV ratio of A/U ≤0.65 (9, 31)].

Normalized penumbra fractions, i.e., Pen_CBF_ and Pen_MTT_ were subsequently calculated as Penumbra volume/(Penumbra volume + Core volume).

### Collateral scores

Collateral scores were independently assessed, without access to clinical information, by a senior neuroradiologist (DJS) with over 10 years of experience in evaluating CTp and CT angiography.

The collateral score used in this study is based on description of angiographic appearance of collateral vessels (32) and applied to CTA maximum intensity projections previously (3, 33–35). We used reconstructed 20 mm axial CTA maximum intensity projections (MIP’s) and assigned collateral scores as follows:

0 = Absent collaterals
1 = Collaterals filling ≤50% of the occluded arterial territory
2 = Collaterals filling >50% but <100% of the occluded arterial territory
3 = Collaterals filling 100% of the arterial territory.

### ASPECTS scoring

**Figure 1:**
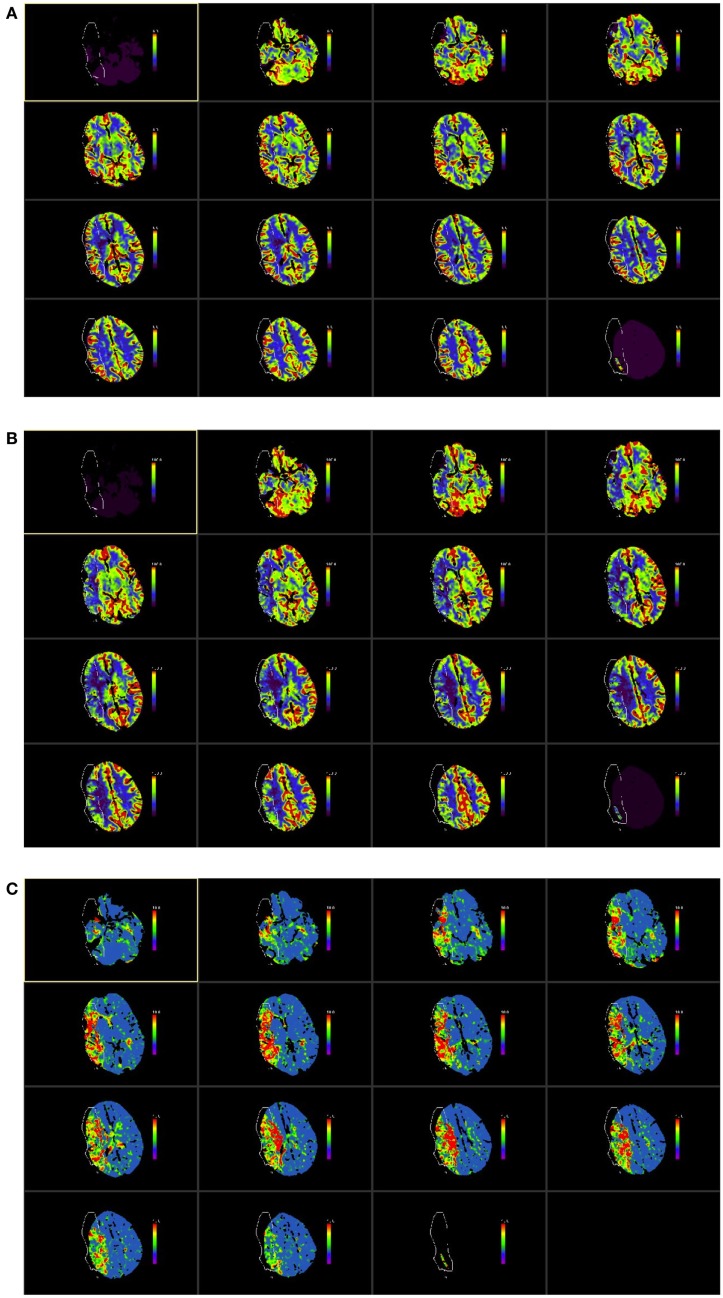
**ASPECTS score was applied CT perfusion maps**. The ASPECTS template divides each hemisphere into 10 vascular regions covering the MCA territory, which include 6 middle cerebral artery cortical regions (M1–M6), caudate nucleus, lentiform nucleus, internal capsule, and insular cortex (23, 24). The CTP maps used were those for cerebral blood volume [CBV **(A)**], cerebral blood flow [CBF **(B)**], and mean transit time [MTT **(C)**] as shown in the illustrative figures below. The images were color scaled, as follows, for each of the parameters consistently across all study subjects: CBV scaled at 0–6 ml/100 ml, CBF color scaled at 0–100 ml/100 ml/s and MTT scaled at 0–10 s. The example in this figure shows a proximal right MCA stroke (outlined in figure). The unaffected hemisphere was used as a reference and each ASPECTS region was compared with the corresponding region on the unaffected hemisphere to assign a score. Each map was scored visually on each of the 10 regions of the ASPECTS template with a score of 0 if the affected side showed a comparative abnormality and a score of 1 if no relative abnormality was seen; thus a total ASPECTS score could vary from 0 to 10 for each of the perfusion maps, with 0 denoting an abnormality across all ten regions and 10 indicating no abnormality in the affected hemisphere. Each rater scored the scans individually and average of the two was subsequently used for the study analysis. In this example, average ASPECTS score was 7 for CBV, 3 for CBF and 2 for MTT.

Two assessors (Tomasz Matys and Smriti Agarwal) independently scored the unthresholded perfusion maps (Figures 1A–C) for each subject. Briefly, ASPECTS score was assigned on a scale of 0–10 for each of the three perfusion maps, a lower score indicating a more extensive perfusion deficit within the stroke lesion. The unaffected hemisphere was used as a reference. While evaluating the maps, raters did not have access to clinical information except for side of the lesion. Individual parameters for each subject were scored on a different calendar day to avoid systematic bias. ASPECTS score for each parameter was averaged from the two readings and this value was used for final analysis.

We evaluated CBF/CBV ASPECTS ratio and MTT/CBV ASPECTS ratio against corresponding penumbra fractions, i.e., Pen_CBF_ and Pen_MTT_, respectively. Where we found a relationship between the two, the corresponding ASPECTS ratio was evaluated against time since onset.

### Statistical analysis

All analyses were performed using IBM SPSS, version 19 for Macintosh and Microsoft Excel 2011. Mean (SD) and median (IQR) values are reported here for baseline clinical characteristics.

For the ASPECTS scoring, interobserver agreement was measured using Kappa statistic (36, 37) and further measure of internal consistency was applied using Cronbach’s alpha (38).

To test our hypothesis, we performed non-parametric correlations using Kendall’s tau-*b* for penumbra fraction, ASPECTS ratios and collateral score against time since stroke onset.

Two sided *p*-values were obtained and considered significant if <0.05.

## Results

### Baseline clinical features

Fifty-three patients were included in this study. Demographic and pertinent clinical data are described in Table 1. Imaging was performed at a mean (SD) time of 125.2 (55.3) minutes from stroke onset. Forty-six patients (86.8%) received intravenous thrombolytic therapy with alteplase. Median (IQR) stroke severity score on the NIHSS (39) was 15 (6). Small vessel disease did not appear in the TOAST classification given that lacunar strokes were excluded. Majority of patients (50.9%) had a cardioembolic etiology for their stroke. About 58.5% of patients had hypertension as a comorbidity and 56.6% had atrial fibrillation.

**Table 1:** **Baseline characteristics (*n* = 53)**.

Mean age in years (SD)	71.3 (14.9)
Sex (M:F)	24:29
Median NIHSS (IQR)	15 (6)
Mean time to imaging in minutes (SD)	125.2 (55.3)
Mean systolic blood pressure (SD)	152.3 (22.2)
Mean diastolic blood pressure (SD)	82.4 (15.7)
Mean blood glucose (SD)	7.6 (1.6)
Mean CRP (SD)	15.3 (29.1)
Mean hematocrit (SD)	0.40 (0.04)
Mean full blood count (SD)	9.5 (3.9)
Mean platelet count (SD)	225.4 (61.7)
Mean body temperature (SD)	36.4 (0.7)
Hypertension *n* (%)	31 (58.5)
History of smoking *n* (%)	24 (45.3)
Current smoking *n* (%)	7 (13.2)
Diabetes mellitus *n* (%)	4 (7.5)
Atrial fibrillation *n* (%)	30 (56.6)
Premorbid antiplatelet therapy *n* (%)	17 (32.1)
Premorbid statin therapy *n* (%)	18 (33.9)
Premorbid antihypertensive therapy *n* (%)	30 (56.6)
Thrombolysis administration *n* (%)	46 (86.8)
Mean premorbid modified Rankin score (SD)	0.4 (0.8)
Mean modified Rankin score at 3 months (SD)	2.4 (2.1)
TOAST classification *n* (%)
Large vessel disease	5 (9.4)
Cardioembolic	27 (50.9)
Other	21 (39.6)

### Penumbra fractions and collateral scores against time since onset

Correlations between penumbra fractions and collateral scores with time since onset are shown in Table 2. Penumbra fraction derived using a CBF threshold, i.e., Pen_CBF_ correlated showing a statistical trend, although non-significantly, with time since stroke onset (Kendall’s tau-*b* = −0.196, *p* = 0.055). Penumbra fraction derived using an MTT threshold, i.e., Pen_MTT_ showed a similar trend (Kendall’s tau-*b* = −0.187, *p* = 0.068).

**Table 2:** **Correlations of penumbra fractions and collateral score with time since stroke onset**.

Parameter	Kendall’s tau-*b*	*p* value
CBF derived penumbra fraction (Pen_CBF_) vs. time since stroke onset	−0.196	0.055
MTT derived penumbra fraction (Pen_MTT_) vs. time since stroke onset	−0.187	0.068
CBF/CBV ASPECTS ratio vs. time since stroke onset	−0.265	0.006
Collateral score vs. time since stroke onset	−0.039	0.724

Collateral score did not correlate with time since stroke onset (Kendall’s tau-*b* = −0.039, *p* value = 0.724).

### ASPECTS score ratio and time since onset

There was significant inter observer agreement based on Fleiss kappa values (36, 37) for ASPECTS scoring and these were 0.581 for CBV (*p* < 0.0001), 0.421 for CBF (*p* = 0.003), and 0.542 for MTT (*p* < 0.0001). As an additional measure of internal consistency, intraclass correlation coefficients were noted in terms of Cronbach’s alpha (38) and these values were 0.739 for CBV, 0.598 for CBF, and 0.713 for MTT indicating that the measurements were reliable and consistent between the two raters.

CBF/CBV ASPECTS ratio showed a positive statistical trend for a relationship with Pen_CBF_ (Kendall’s tau-*b* = 0.190, *p* value = 0.070); MTT/CBV ASPECTS did not show a relationship with Pen_MTT_ (Kendall’s tau-*b* = −0.094, *p* value = 0.368). CBF/CBV ASPECTS ratio was thus, used as a surrogate for the quantitative penumbra fraction, so simple visual assessment of perfusion maps could be examined against time since onset.

There was a significant inverse relationship between ASPECTS CBF/CBV against time since stroke onset (Kendall’s tau-*b* = −0.265, *p* value = 0.006) as shown in Table 2.

**Figure 2:**
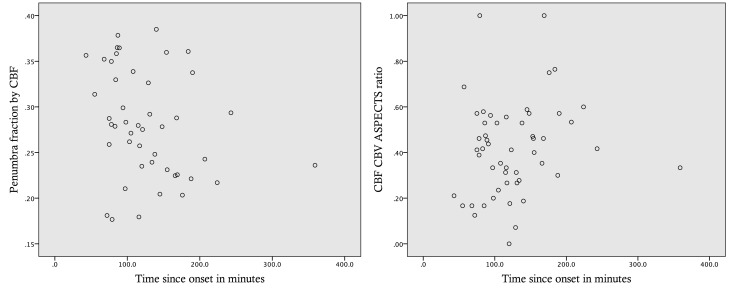
**Perfusion parameters and time since stroke onset**. Scatter plots for penumbra fraction defined by a CBF threshold (Pen_CBF_) and CBF/CBV ASPECTS ratios against time since stroke onset.

Figure 2 shows the relationship between Pen_CBF_ ratio and time, and between CBF/CBV ASPECTS ratio and time.

## Discussion

Our quantitative analysis, using CBF and MTT thresholds derived from published literature, showed a trend for a relationship of penumbra fraction with time, in the expected negative direction. Toward an application in the wider clinical setting, we also investigated visual assessment of perfusion abnormalities, applying the previously validated ASPECTS approach (23, 40) to CTp. Interobserver agreement and internal consistency measures for the two raters were in keeping with published literature (24). At variance with the quantitative analysis, the visual analysis using the CBF/CBV ASPECTS ratio showed a statistically significant decremental relationship over time. Larger studies are warranted to confirm these visual assessment-based findings and explore clinical applicability in detail.

MRI-based timing of stroke lesions has been previously investigated in detail, with DWI-FLAIR mismatch being a predictor of stroke onset within 4.5 h (36), leading on to an ongoing clinical trial (41). While MRI-based methods have been more widely studied (36), perfusion CT-based evaluations are limited (42, 43). Given the ease of access, shorter scanning times, lower cost, and less susceptibility to movement artefacts (11, 44), CTp has potential clinical utility and has been successfully compared to MRI-based methods in acute stroke (45).

One potential issue with using quantitative thresholds to identify the penumbra and core using CTp that could account for our marginal findings, is the lack of formal validation so far, resulting in various groups using different data processing and perfusion variables and thresholds (10, 46). Generally, penumbral imaging holds potential promise to clinical translation, although a number of early trials of treatments using these methods have been negative (47). There are a number of limitations of these studies including methodological variability and lack of evaluation in an early time window due to previous evidence being based on plain CT imaging. More recently, two positive trials used CTp and quantitative perfusion thresholds to select optimal candidates to evaluate new thrombolytic agents (20) and endovascular intervention (21), may lead to changes in practice in due course.

Our data show that collateral response does not change over time in the early window we studied. One explanation is that collateral response may be intrinsically variable in individuals with proximal occlusions and thus, either present or not (48). It is in turn feasible that the collateral status affects the relationship between the penumbra ratio and time, complicating the across-subject relationship. For instance, one would expect that if there are good collaterals, the ratio would remain higher for a longer period, until the penumbral tissue has exhausted its energy reserve and proceeds to infarction (13, 14). Thus, the difference in collaterals between individuals may explain why time only trended toward association with penumbra ratio, as the rate of conversion to an ischemic core is dependent on not only time, but also the presence or absence of efficient collaterals in any given individual. Our small sample size precludes a meaningful multivariate analysis to answer these questions. Future studies with larger patient populations are thus needed.

Thus far published CT-based characterization of “wake up/strokes of unclear onset” have not been able to identify any specific features compared to those events where the time of onset is known (33, 35, 49, 50), with the exception of one study that found higher frequency of hypodensity on non-contrast CT in the “wake up/unclear onset” group (33). Heterogeneity in time since onset may be one reason. There is some evidence in clinical studies that stroke on awakening may develop shortly prior to presentation unlike unwitnessed stroke due to other reasons (33, 51) and thus, at least in a subset of patients where time of symptom onset is not known, CTp parameters that we describe may have role. However, we acknowledge that the lack of patients beyond the 4.5-h window is a clear limitation of our study with respect to wake up/unknown time of onset strokes. Larger studies with patients beyond this time window may help confirm the trends we demonstrate in our quantitative data and ASPECTS derived metrics. We studied patients in the time window for current thrombolysis license (52) due to the observational nature of our study. However, given the current clinical evidence-based guidelines or thrombolysis in stroke cover the first 4.5 h post ictus, our study may also have utility in this very clinical setting.

Another confound of our study, which may explain why we were unable to detect statistically significant relationships in our quantitative data, is the small sample size. Larger datasets of patients in longer time windows are needed to confirm the trends that we demonstrate, both, with respect to the quantitative findings and the visual assessment-based findings. We also recognize that the visual assessment approach could be improved further, particularly for CBF, while future studies will need to assess optimal thresholds when applying the quantitative method, which may provide more reliable clinically applicable indices.

## Conclusion

In this pilot study of patients with proximal arterial occlusions, we find some evidence towards a relationship between CTp and time since onset within the currently licensed thrombolysis window, which if confirmed in larger studies with broader inclusion times, could have implications in the clinical setting of strokes of unknown onset time.

## Conflict of Interest Statement

The authors declare that the research was conducted in the absence of any commercial or financial relationships that could be construed as a potential conflict of interest.
